# Metachronous Testicular Cancer After Orchiectomy: A Rare Case

**DOI:** 10.7759/cureus.1833

**Published:** 2017-11-09

**Authors:** Ersan Arda, Basri Cakiroglu, Gizem Cetin, Ilkan Yuksel

**Affiliations:** 1 Urology, Trakya University Medical Faculty; 2 Urology, Hisar Intercontinental Hospital; 3 Anesthesiology, Trakya University Medical Faculty; 4 Urology, Private Bir Nefes Hospital

**Keywords:** testicular cancer, orchiectomy, intratubular germ cell

## Abstract

Testicular cancer represents approximately 1% of all cancers diagnosed in males. The prevalence of bilateral testicular germ cell tumor cases varies from 1% to 5%. Intratubular germ cell neoplasia (ITGCN) is a precursor for almost all testicular germ cell tumors (TGCT) and is one of the highest risks of developing contralateral testicular cancer. The radical orchiectomy is still preferred for the treatment of testicular cancer. However, in some cases like solitary testis, bilateral cancer or if the tumor size is under 30% percent of the testicular extent, organ-sparing surgery can be an option. There are just a few published reports of metachronous contralateral testicular cancer, developed after orchiectomy with the histopathology of the intratubular germ cell neoplasia.

## Introduction

Testicular cancer is the most commonly diagnosed cancer in young males. In the United States, according to the Cancer Society statistics of 2015, approximately 8430 new testis cancer patients have been diagnosed [1]. The testicular germ cell tumors (TGCTs) are 90-95% of testicular tumors, which can be mainly divided into seminomas and non-seminomas histopathologically.

According to published large series, the prevalence of bilateral TGCTs cases varies from 1%- 5%, which among them the metachronous tumors are the most common type with 65% [2-5]. It is well established that intratubular germ cell neoplasia (ITGCN) is a precursor for almost all TGCTs and is one of the highest risks of developing contralateral testicular cancer, with up to 25 times, in the history of TGCTs [6-7].

However, there are only just a few reported cases with metachronous contralateral testicular cancer, developed after an orchiectomy with the histopathology of ITGCN.

## Case presentation

We report a case of a 28-year-old male who underwent a left radical orchiectomy for an ultrasonographically proved painless left testicular mass, six years ago. At that time, the testicular tumor markers alpha-fetoprotein (AFP), beta human chorionic gonadotropin (bHCG), lactate dehydrogenase (LDH) were negative and the computed tomography (CT) scan didn’t show any evidence of the metastatic lesion. The pathological evaluation demonstrated a 4.5x3x3 cm sized teratoma including focal immature ranges with ITGCN limited to the testis.

The patient was followed up with the physical examination, tumor marker assessments, chest x-ray and CT scan based on the European Urological Association guidelines. After four years of the disease-free period, an approximately 1.5 cm right testicular mass was identified during physical examination during a routine outpatient clinic visit. The testis-sparing surgery was performed. The pathologic evaluation of the specimen revealed a 60% embryonal carcinoma and 40% teratocarcinoma. Although the tumor marker levels were normal and radiologic findings were free of metastasis, two doses of chemotherapy (bleomycin-etoposide cisplatin) were given to the patient.

Approximately two years after the chemotherapy, the patient appealed with right testicular pain and tenderness. The Scrotal Doppler ultrasonography revealed two hypoechoic nodular lesions at the central zone with 3x6 mm and 5x7 mm in size. The Scrotal magnetic resonance imaging (MRI) demonstrated right testicular lesions with 9x7 mm and 4x4 mm in size. The serum concentrations of lactate dehydrogenase (LDH), beta human chorionic gonadotropin (bHCG) and alpha-fetoprotein (AFP) tests were evaluated as 227 U/L (135-225), 6,7 mIU/mL(0-2) and 5,8 ng/mL (0-7) respectively.

After the patient was informed about possible management options, right radical orchiectomy and placement of bilateral testicular prosthesis were performed. The final histopathologic diagnosis was reported as teratocarcinoma. The hormone replacement was carried out onset with daily topical testosterone, postoperatively. At the first follow up, the patient was free of disease and no adverse effects due to the replacement therapy were established.

## Discussion

Testicular cancer represents approximately 1% of all the cancers diagnosed in the males [2]. Nearly 50% of the males are developing invasive cancer after ITGCN within five years, especially before 60 months when the second tumor is commonly associated with seminomatous histopathology [7-8].

According to the literature, the incidence of ITGCN in younger males is under 1% and mostly unilateral [6,9]. In European centers where a contralateral testicular biopsy is routinely performed, the prevalence of ITGCN was found to be 4-5, 4% and the risk of developing a subsequent invasive tumor, in patients with TGCTs was 3, 4-4% in the patients with TGCTs [8-9]. Due to long-term follow-up of the patients with treated testicular cancer, researchers reported 67% metachronous contralateral tumors within five years after the first diagnosis. There are very rare published case reports of metachronous contralateral testicular cancer, developed after an orchiectomy with the histopathology of ITGCN.

The radical orchiectomy is still preferred for the treatment of testicular cancer and ITGCN with normal contralateral testis, but in some cases like solitary testis, bilateral cancer or if the tumor size is under 30% percent of testicular extent, the organ-sparing surgery can be an option [10].

Additionally, to the recommended treatment for ITGCN which is radical surgery or low dose radiotherapy, platinum-based chemotherapy has been suggested to be effective, but the response is still controversial due to unproofed diagnosis on follow-up biopsies [10]. Our patient underwent two doses of bleomycin-etoposide cisplatin protocol, but approximately two years after the chemotherapy, the recurrence was observed. It is suggested that first-time tumor histology, receiving chemotherapy, and/or the period between metachronous tumors are factors that may influence the histology of a second tumor.

Finally, bilaterally orchiectomy testicular cancer patients need a whole-life hormone replacement therapy. There is the need for the testosterone substitution in order to minimize adverse effects or risks associated with hypogonadism like metabolic syndrome, diabetes and/or cardiovascular diseases.

## Conclusions

The incidence of ITGCN in younger males is under 1% and mostly unilateral. However, every patient with testicular cancer has an increased risk of developing a contralateral testicular malignancy, even years after the diagnosis or after the first treatment.
